# First molecular investigation of *Cryptosporidium* spp. in young calves in Algeria

**DOI:** 10.1051/parasite/2017014

**Published:** 2017-05-12

**Authors:** Djahida Benhouda, Ahcène Hakem, Anna Rosa Sannella, Afaf Benhouda, Simone M. Cacciò

**Affiliations:** 1 Laboratory of Exploration and Valorization of Steppic Ecosystems, Faculty SNV, University of Ziane Achour 17000 Djelfa Algeria; 2 Department of Infectious Diseases, Istituto Superiore di Sanità 00161 Rome Italy; 3 Biotechnology Laboratory of Bioactive Molecules and Cellular Physiopathology, Faculty of Biological Sciences, Department of Living Organisms, University of Batna 2 05000 Batna Algeria

**Keywords:** *Cryptosporidium*, Cryptosporidiosis, Molecular characterization, Cattle, Algeria

## Abstract

To date, no information is available on the prevalence and genetic identity of *Cryptosporidium* spp. in cattle in Algeria. In this study, 17 dairy farms in the province of Batna, located in the northeast of the country, were visited to collect 132 fecal samples from young calves (< 8 weeks old). Samples were examined microscopically using the modified Ziehl-Neelsen acid-fast staining method, and at least one sample per farm was submitted for molecular analysis. Amplification of a fragment of the small subunit ribosomal RNA gene was positive for 24 of the 61 samples (40%), and sequence analysis identified three species, namely *Cryptosporidium bovis* (*n* = 14), *C. ryanae* (*n* = 6), and *C. parvum* (*n* = 4). The *C. parvum* IIaA13G2R1 subtype, an uncommon zoonotic subtype, was identified in two isolates from a single farm by sequencing a fragment of the GP60 gene. This is the first report about genotyping and subtyping of *Cryptosporidium* in calves in Algeria.

## Abstract (fr)

Actuellement, aucune information n’est disponible sur la prévalence et l’identité génétique de *Cryptosporidium* spp. chez les bovins en Algérie. Dans ce travail, 17 fermes laitières de la province de Batna, située au nord-est du pays, ont été inspectées pour récolter 132 échantillons fécaux de jeunes veaux (< 8 semaines). Les échantillons ont été examinés microscopiquement en utilisant la méthode de Ziehl–Neelsen modifiée, et au moins un échantillon par ferme a été soumis à analyse moléculaire. L’amplification d’un fragment du gène de la petite sous-unité du RNA ribosomal a été positive pour 24 échantillons parmi 61 (40 %), et l’analyse des séquences a identifié trois espèces, *Cryptosporidium bovis* (*n* = 14), *C. ryanae* (*n* = 6) et *C. parvum* (*n* = 4). Le sous-type IIaA13G2R1 de *C. parvum*, un sous-type zoonotique peu répandu, a été identifié par séquençage d’un fragment du gène GP60 dans deux isolats d’une seule ferme. Ceci est le premier rapport sur le génotypage et le sous-typage de *Cryptosporidium* chez les veaux en Algérie.

## Introduction

Parasites of the genus *Cryptosporidium* cause diarrheal disease in many vertebrate hosts, including humans, and have a worldwide distribution [6, 7]. Currently, 31 *Cryptosporidium* species have been recognized based on biological and molecular characteristics, while many other genotypes are still of uncertain taxonomic status [21]. In humans, infection is mostly caused by two species, *Cryptosporidium hominis* and *C. parvum*; the former is considered restricted to humans, whereas the latter also infects other mammals, in particular young ruminants. Other species commonly occur in cattle, including *C. bovis*, *C. ryanae*, and *C. andersoni* [22]. The distribution of these species is age-dependent: *C. parvum* predominates in pre-weaned animals, whereas *C. bovis* and *C. ryanae* are more common in post-weaned animals and young stock, while *C. andersoni* is mostly found in adult cattle [11]. Therefore, pre-weaned calves are considered important reservoirs of *C. parvum* oocysts infectious to humans, and outbreaks associated with exposure to calf feces are well documented [13].

In Africa, cryptosporidiosis is a particularly relevant health problem, and recent studies have shown that *Cryptosporidium* is second only to Rotavirus among etiological agents of moderate to severe diarrhea in very young children [17]. Many studies have been conducted in African countries to estimate the burden of cryptosporidiosis in humans and animals, describe the circulating species and genotypes, and elucidate transmission routes [1, 8]. However, no information is currently available on human cryptosporidiosis in Algeria, and only a few studies have been conducted on animals, with a focus on horses and donkeys [18], and broiler chickens and turkeys [3].

Here, we provide the first evidence of *Cryptosporidium* spp. infection in young calves (<2 months) from small, traditional farms in Algeria. We performed molecular investigations to define the parasite species.

## Materials and methods

### Collection of samples

**Figure 1.**
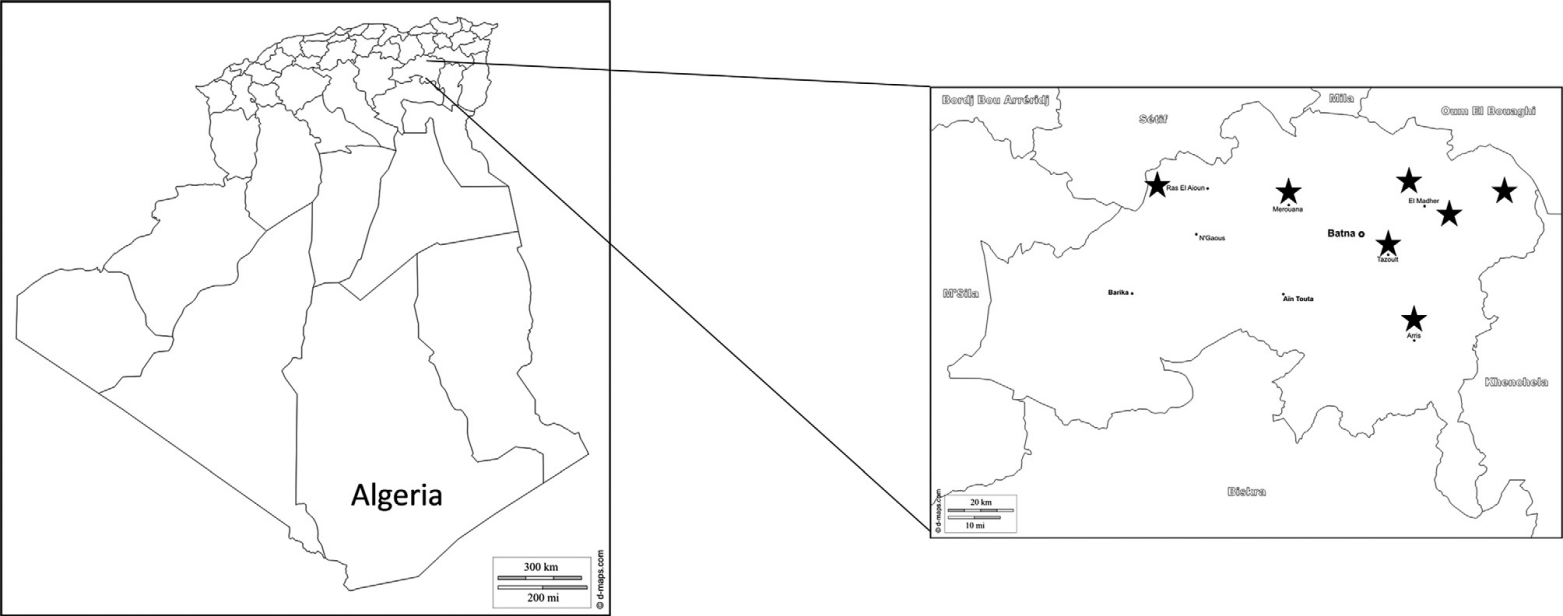
Map of Algeria and of the Batna province showing the location of the dairy farms investigated in this study. The original maps were downloaded from http://www.d-maps.com/carte.php?num_car=176844&lang=en

The study was carried out in the Wilaya (province) of Batna, situated in the northeast of Algeria (Figure 1). This province covers about 12,192 km^2^ and has a population of approximately 1,120,000 inhabitants. During May 2016, a single visit was conducted at 17 dairy farms, and 132 samples were collected. These are traditional farms with a small number of adult cattle (from 6 to 40; age 2–9 years), mostly of the local breed known as *Race Brune de l’Atlas*. Calves are usually reared indoors with their mothers and fed with bottled milk.

A minimum of 5 g of feces was collected from each pre-weaned or post-weaned calf, either directly from the rectum, when possible, or from freshly deposited feces on the ground. Each sample was individually placed into a sterile plastic tube, mixed with an equal volume of 5% potassium dichromate, and transported to the laboratory of parasitology in a refrigerated box.

Fecal specimens were screened by microscopy for *Cryptosporidium* oocysts after staining with the modified Ziehl-Neelsen stain [5]. A semi-quantitative score was used to distinguish between low (1–4 oocysts per field), moderate (5–10 oocysts per field), and high (>10 oocysts per field) levels of infection.

### DNA isolation and molecular analysis

Fecal specimens were washed three times with distilled water by centrifugation to remove potassium dichromate prior to DNA isolation. Total DNA was extracted from ~200 mg of feces using a commercially available kit (QIAamp^®^ DNA Stool Mini Kit, Qiagen, Hilden, Germany) in accordance with the manufacturer’s instructions. Purified DNA was stored at −20 °C prior to Polymerase Chain Reaction (PCR).

For species identification, a nested PCR assay was used to amplify a ~590 bp fragment of the small subunit rRNA (SSU rRNA) gene [20]. For subtyping, a ~300 bp fragment of the glycoprotein 60 (GP60) gene, encompassing the microsatellite region at the 5′ of the gene, was amplified by nested PCR [9]. Negative and positive controls (DNA from the *C. parvum* Moredun strain) were included in each experiment. PCR was performed using 25 μL of 2× GoTaq Green (Promega, Madison, WI, USA), 10 pmol of each primer, 2.5–5.0 μL of DNA, and nuclease-free water up to a final volume of 50 μL. Reactions were performed on a Perkin Elmer 9700 apparatus (Life Technologies, Carlsbad, CA, USA). Aliquots (5–10 μL) of the PCR products were loaded on 1.5% agarose gel stained with ethidium bromide. PCR products were purified using spin columns (Qiaquick PCR Purification kit, Qiagen, Milan, Italy) and sequenced directly on an ABI 3130 Genetic Analyzer. Bidirectional sequences were edited and assembled using the SeqMan 7.1 software package (DNASTAR, Madison, WI, USA). A BLAST search against the GenBank database was used to identify *Cryptosporidium* species and subtypes.

New sequences were deposited in GenBank with Accession Numbers KY765343, KY765344, KY765345, and KY775519.

## Results

### *Cryptosporidium* species in cattle

Based on microscopic analysis of fecal smears stained using the modified Ziehl-Neelsen acid-fast method, the majority of the samples (84%) at each of the 17 investigated farms were positive (Table 1), suggesting a herd prevalence of 100%. In the majority of the samples (58%), however, few oocysts were observed. Due to the possibility of false negative results of microscopy, and the higher sensitivity of PCR-based procedures, a panel of 66 samples, comprising at least one sample per farm, was selected for molecular analysis. Nested PCR amplification of a fragment of SSU rRNA resulted in the identification of 24 positive samples (36%). At the farm level, 14 of 17 had at least one sample positive by PCR (Table 1), suggesting a prevalence of 82% at the herd level and of 18% (24 of 132) at the animal level.

**Table 1.** *Cryptosporidium* species identified in dairy cattle in Algeria.

Location of farm	Farm ID	Cattle at farm	Fecal samples collected	Positive/tested by microscopy	Positive/tested by PCR	Species identified (number)
Djarma	1	33	7	7/7	2/7	*Cryptosporidium bovis* (1)
						*Cryptosporidium ryanae* (1)
	2	6	2	2/2	½	*Cryptosporidium bovis* (1)
	17	6	2	2/2	2/2	*Cryptosporidium bovis* (2)
Ain yagout	3	26	10	10/10	3/7	*Cryptosporidium bovis* (3)
	4	40	10	10/10	3/5	*Cryptosporidium bovis* (2)
						*Cryptosporidium parvum* (1)
Maader	5	20	6	6/6	4/6	*Cryptosporidium bovis* (2)
						*Cryptosporidium parvum* (2)
Arris	6	7	3	3/3	0/3	
	7	10	8	8/8	1/2	*Cryptosporidium ryanae* (1)
Marwana	8	40	8	8/8	2/3	*Cryptosporidium parvum* (1)
						*Cryptosporidium ryanae* (1)
Chemora	9	15	5	4/5	1/5	*Cryptosporidium ryanae* (1)
	10	10	4	4/4	1/1	*Cryptosporidium ryanae* (1)
	11	11	5	3/5	0/5	
Bouhmar	12	34	24	15/24	1/5	*Cryptosporidium bovis* (1)
	13	27	9	9/9	1/1	*Cryptosporidium bovis* (1)
Raas Laayeon	14	11	5	2/5	0/5	
	15	28	15	11/15	1/4	*Cryptosporidium bovis* (1)
	16	32	9	7/9	1/3	*Cryptosporidium ryanae* (1)
Total			132	111/132	24/66	

Sequencing of the SSU rRNA amplicons identified *Cryptosporidium bovis* as the most common species (*n* = 14, present in nine farms), followed by *C. ryanae* (*n* = 6, present in six farms) and *C. parvum* (*n* = 4, present in three farms). In particular, the sequences obtained had 100% similarity to GenBank reference sequences for *C. bovis* (AY741305), *C. ryanae* (EU410344), and *C. parvum* (KY514062). No intra-species sequence variation in the SSU rRNA gene fragment was observed.

*Cryptosporidium bovis* was more common in calves aged 1–2 months (*n* = 11) than in those aged 15–20 days (*n* = 3); likewise, *Cryptosporidium ryanae* was found in five calves aged 1–2 months but only in a single 15-day-old calf. Finally, *Cryptosporidium parvum* was found only in calves younger than 1 month.

### 3.2 *Cryptosporidium* subtypes in cattle

Sequencing of a fragment of the GP60 locus revealed subtype IIaA13G2R1 in two animals from the same farm (Table 1). The sequences had 100% similarity to GenBank sequence KF008184.

## Discussion

In a global African perspective, human-to-human transmission of *Cryptosporidium* is considered the main route of transmission, at least in Sub-Saharan countries, where *C. hominis* and anthroponotic (human host-restricted) subtypes of *C. parvum* account for the vast majority of cases observed in young children [24]. Nevertheless, cryptosporidiosis is also common in a range of domestic and wild animal species, and evidence for zoonotic potential has been provided in many studies [1, 14].

In this context, molecular studies on human and animal cryptosporidiosis in North African countries are still scarce. In Egypt, calves are predominantly infected with *C. parvum* subtypes of the IId and IIa families [2], which are also found in humans in this country [15]. In Tunisia, *C. parvum* IIaA15G2R1 and IIdA16G1 subtypes were identified in calves and children from a rural area in the north of the country [19]. Another study identified *C. hominis*, *C. parvum*, and *C. meleagridis* in immunocompetent and immunocompromised individuals, mostly children, in Tunisia [10]. Therefore, both zoonotic and anthroponotic species are involved in human cryptosporidiosis in these regions.

Here, we provide the first information on the prevalence and genetic identity of *Cryptosporidium* species in young calves (<2 months) reared in small, traditional farms in the north of Algeria. Our data show that calves aged 1–2 months are mostly infected with *Cryptosporidium bovis* followed by *C. ryanae*, whereas few animals were infected with *C. parvum*. This contrasts with the prevailing pattern of *C. parvum* dominance in young calves [22], but is consistent with data from less intensive management systems in different parts of the world [4, 23, 27], where *C. bovis* is the dominant species even in pre-weaned calves.

Four calves from three farms were positive for *Cryptosporidium parvum*, and subtyping of the GP60 gene in two isolates from a single farm identified subtype IIaA13G2R1. This uncommon subtype has been found in calves in Turkey [25], Canada [26], Belgium [12], and the Netherlands [28], and in people with HIV/AIDS in Malaysia [16] and in the United States [28].

In conclusion, the data presented suggest that cattle play a minor role in sustaining circulation of zoonotic *Cryptosporidium* species/genotypes. However, a better estimate of the prevalence and identity of the *C. parvum* genotypes in young calves, and a clarification of their role in clinical cryptosporidiosis are needed. Likewise, understanding the relative role of anthroponotic and zoonotic transmission in Algeria will require investigations into human cryptosporidiosis.

## Conflict of interest

The authors have no competing interests.
